# Impact of Weaning and Maternal Immune Activation on the Metabolism of Pigs

**DOI:** 10.3389/fmolb.2021.660764

**Published:** 2021-07-15

**Authors:** Bruce R. Southey, Courtni R. Bolt, Haley E. Rymut, Marissa R. Keever, Alexander V. Ulanov, Zhong Li, Laurie A. Rund, Rodney W. Johnson, Sandra L. Rodriguez-Zas

**Affiliations:** ^1^ Department of Animal Sciences, University of Illinois at Urbana-Champaign, Urbana, IL, United States; ^2^ Roy J. Carver Biotechnology Center, University of Illinois at Urbana-Champaign, Urbana, IL, United States; ^3^ Neuroscience Program, University of Illinois at Urbana-Champaign, Urbana, IL, United States; ^4^ Illinois Informatics Institute, University of Illinois at Urbana-Champaign, Urbana, IL, United States; ^5^ Department of Statistics, University of Illinois at Urbana-Champaign, Urbana, IL, United States

**Keywords:** gas chromatography, mass spectrometry, maternal immune activation, weaning, hepatic metabolomics stress

## Abstract

Weaning wields environmental, social, and nutritional stresses that are detectable in the blood metabolite levels of the offspring. Prenatal stress in the form of maternal immune activation (MIA) in response to infection, which is associated with health and behavior disorders, also elicits prolonged changes in blood and brain cytokine and metabolite levels of the offspring. The goal of this study was to investigate the effects of weaning and MIA on the offspring’s liver function to advance the understanding of the impact of stressors on peripheral and central nervous systems, physiology, and health. Gas chromatography–mass spectrometry analysis was used to compare the level of hepatic metabolites from 22-day-old pigs (*n* = 48) evenly distributed among weaning (nursed or weaned), viral MIA exposure (yes or no), and sexes. Weaning effects were detected on 38 metabolites at *p*-value < 0.05 (28 metabolites at FDR *p*-value < 0.05), and sex-dependent MIA effects were detected on 11 metabolites. Multiple intermediate and final products of the enriched (FDR *p*-value < 0.05) glycolysis and gluconeogenesis and pentose phosphate pathways were over-abundant in nursed relative to weaned pigs. The enriched pathways confirm the impact of weaning on hepatic metabolic shift, oxidative stress, and inflammation. Higher levels of the glucogenic amino acid histidine are observed in pigs exposed to MIA relative to controls, suggesting that the role of this metabolite in modulating inflammation may supersede the role of this amino acid as an energy source. The lower levels of cholesterol detected in MIA pigs are consistent with hypocholesterolemia profiles detected in individuals with MIA-related behavior disorders. Our findings underline the impact of weaning and MIA stressors on hepatic metabolites that can influence peripheral and central nervous system metabolic products associated with health and behavior disorders.

## Introduction

The process of weaning exerts nutritional, physiological, environmental, and social stresses on the offspring (Campbell et al., 2013). Rodent and pig models demonstrate that shortly after weaning, the immune status of the offspring is low while transitioning from the passive immunity offered by the mother’s milk to developing individual active immunity. During this period, the offspring is susceptible to infection, can experience digestive problems due to the diet change, and must adapt to a new environment and possibly interactions with new pen or cage mates (Jayaraman and Nyachoti, 2017; Xiong et al., 2019). Weaning activates the hypothalamus–pituitary–adrenal (HPA) axis and then increases the level of circulating stress hormones (Moeser et al., 2017).

The nutritional, hormonal, and inflammatory changes triggered by weaning stress can influence metabolic processes and organ function (Le Dividich and Seve, 2000). For example, glucose is processed into fat or glycogen and, through gluconeogenesis, these can be back-transformed into glucose in the liver. In addition to glycogen, the liver produces lipids, cholesterol, and albumin and stores iron and vitamins. The liver also filters toxins and chemicals from the blood and reprocesses hemoglobin.

Weaning stress can result in hepatic dysfunction, hindering the pig’s health and growth. Within 5–8 h after weaning, the pig undergoes a period of fasting or restricted intake (Le Dividich and Seve, 2000; Le Dividich and Sève, 2001), and the liver produces glucose initially by gluconeogenesis before using and other sources such as lactate and amino acids (Han et al., 2016). Weaning stress can create a circular response where stress reduces feed intake that leads to poorer gut health and an increased immune response which in turn leads to reduced feed intake and further liver dysfunction (Jayaraman and Nyachoti, 2017).

Hormone output from the HPA axis and inflammatory signals influence hepatic function (Genes, 1977). Cytokines can mediate hepatic inflammation and degeneration (i.e., apoptosis, necrosis, cholestasis, and fibrosis) and also regeneration (Tilg, 2001). Alteration of the levels of cytokines interleukin (IL)-3 and -6 in response to immune stressors can disrupt gluconeogenesis and other liver metabolic processes. More than 20 cytokines including tumor necrosis factor (TNF) *α* (TNF-α), growth factors, and hormones activate the Janus kinase–signal transducer and activator of transcription (JAK–STAT) signaling pathway, and these mechanisms are associated with liver inflammation, injury, and repair (Gao, 2005).

Gestating females can develop an immune response to infection and other stressors, akin to the inflammatory signals elicited by postnatal stressors. Maternal immune activation (MIA) can in turn elicit an immune response in the fetus that can impair neurodevelopment and have long-term effects on the offspring. The effects of MIA have been associated with behavior disorders (Rymut et al., 2020), autism spectrum disorder, and schizophrenia spectrum disorders. The molecular mechanisms underlying neurological disorders are starting to be characterized (Antonson et al., 2017; Keever et al., 2020; Rymut et al., 2020). Pig and rodent fetuses’ response to maternal immune response includes activation of inflammatory cytokines and structural and functional dysregulation of developing central nervous system regions (Schaafsma et al., 2017).

The inflammatory response of gilts infected with porcine reproductive and respiratory syndrome virus (PRRSV), a member of the Arteriviridae family within the same Nidovirales order that includes the Coronaviridae family (Enjuanes et al., 2008), during gestation was associated with changes in locomotion and sickness behavior of 60-day-old pigs (Keever et al., 2020) and social behavior changes in 21- to 27-day-old pigs (Antonson et al., 2017; Rymut et al., 2020). At molecular levels, the MIA elicited by PRRSV was associated with changes in the expression of genes annotated to immune response in the amygdala of 22-day-old pigs in both a sex-dependent and a sex-independent manner (Keever et al., 2020) and increases in the expression of TNF-α and interferon-γ genes in the fetal hippocampus (Antonson et al., 2018).

The established effects of MIA on the hippocampus and amygdala may extend into the liver. The hippocampus modulates the activity of the HPA, and therefore, the MIA effects on fetal hippocampal cytokine pathways can have downstream effects on the HPA neuroendocrine activity and ultimately affect liver function (Lathe, 2001). Likewise, studies suggest that the amygdala may modulate the regulation of hepatic glucose metabolism and thereof systemic glucose levels, directly or through projections into the hypothalamus or other brain structures (Aizawa et al., 2004; Boghossian et al., 2009; Posey et al., 2009; Soto et al., 2019).

Although studies of the impact of MIA on the offspring have focused on the central nervous system, the impact of MIA on the gastrointestinal systems is starting to be characterized. A study of rats challenged with lipopolysaccharide (LPS) during gestation demonstrated that maternal immune stimulation affected the offspring’s hepatic inflammatory response mediators (Surriga et al., 2009). The liver of pups born from LPS-challenged dams had lower expression levels of the IL-6 gene and lower phosphorylation of the extracellular signal–regulated kinase p42/44 MAPK complex that participates in the regulation of IL-6 expression in hepatocytes.

We postulate that hepatic function can be affected by molecular signals elicited by weaning stress. Moreover, prenatal MIA may also disrupt the offspring’s hepatic metabolism, and these effects can be long-lasting. The objective of this study is to advance the understanding of the effects of weaning stress and MIA, acting alone or in combination, on the hepatic metabolism of pigs. A supporting objective was to characterize potential sex-dependent effects of weaning stress and MIA. An analysis of metabolomic profiles using gas chromatography–mass spectrometry was undertaken. Alterations in metabolism product levels can assist in the development of treatments and management practices that minimize hepatic dysfunction associated with weaning and MIA stressors.

## Materials and Methods

### Animal Experiments

All experimental procedures were approved by the Illinois Institutional Animal Care and Use Committee (IACUC) at the University of Illinois at Urbana-Champaign and comply with the USDA Animal Welfare Act and the NIH Public Health Service Policy on the Humane Care and Use of Animals.

Metabolomic profiles were studied in 22-day-old pigs evenly distributed among weaning groups (nursed and weaned), sexes (females and males), and whether exposed or not exposed to PRRSV-elicited MIA. The experiment encompassed 48 pigs in a 2 × 2 × 2 design with *n* = 6 pigs per MIA–weaning–sex group (Keever et al., 2020; Rymut et al., 2020). Briefly, the pigs studied were offspring from Camborough gilts (PIC, Hendersonville, TN) from the University of Illinois Swine Research Center herd that were inseminated with PIC 359 boar semen (PIC, Hendersonville, TN). At gestation day 69, PRRSV-negative gilts were individually housed in identical disease containment chambers maintained at 22°C with a 12-h light/dark cycle with lights on at 7:00 AM following proven protocols (Antonson et al., 2017; Keever et al., 2020; Rymut et al., 2020).

The gilts (*n* = 12) were randomly assigned into two MIA groups, maternal PRRSV activated (MPA group) or control gilts (CON group). Corresponding to the initiation of rapid fetal brain growth, MPA gilts were intranasally inoculated at gestation day 76 with the live PRRSV strain P129-BV (School of Veterinary Medicine at Purdue University, West Lafayette, IN) at a dose of 1 × 10^5^ 50% tissue culture infectious dose (TCID_50_) diluted in sterile Dulbecco’s modified Eagle’s medium (5 ml total volume), whereas CON gilts received 5 ml total volume of sterile Dulbecco’s modified Eagle’s medium (Antonson et al., 2017; Keever et al., 2020; Rymut et al., 2020). The gilts were fed a gestational diet of 2.3 kg per day and had *ad libitum* access to water throughout the experiment. After PRRSV inoculation, CON gilts were fed the average consumption of the MPA gilts on the preceding day. One week after inoculation, multiplex PRRSV real-time reverse transcription polymerase chain reaction saliva tests (Veterinary Diagnostic Laboratory, College of Veterinary Medicine, University of Illinois at Urbana-Champaign, Urbana, IL) demonstrated that all MPA gilts were positive for PRRSV and all CON gilts were negative for PRRSV. MPA gilts exhibited a significant increase in rectal temperatures and a decrease in feed intake within 48 h of inoculation, and rectal temperatures and feed intake returned to baseline levels by 14 days (Keever et al., 2020).

On gestation day 113, farrowing was induced using an intramuscular injection of 10 mg of Lutalyse (Pfizer, New York, NY). At birth, the pigs were given intramuscular injections of iron dextran (100 mg/pig, Butler Schein Animal Health, Dublin, OH) and penicillin (60 kU/pig, Butler Schein Animal Health, Dublin, OH) (Keever et al., 2020). The pigs remained with the gilts in individual farrowing crates of standard dimensions (1.83 × 1.83 m). While nursing, the pigs had access to the gilt’s feeder. Water remained available *ad libitum* for the duration of the experiment. At 21 days of age, pigs were randomly selected within MIA and sex into two groups. One group were weaned (WEA group; *n* = 24), and the other group remained with sow nursing (NUR group; *n* = 24). Pigs in the WEA group were group-housed (four pigs per pen) receiving a nutritionally complete diet for growing pigs and *ad libitum* access to water (Antonson et al., 2017; Keever et al., 2020; Rymut et al., 2020). By day 21, the pigs exposed to MIA were 1.20 kg lighter than the pigs not exposed to MIA, yet the difference was not statistically significant (*p*-value < 0.089), and there was no significant difference in body weight between nursed and weaned pigs (Keever et al., 2020).

At 22 days of age, all pigs were anesthetized intramuscularly using a telazol:ketamine:xylazine drug cocktail (50 mg of tiletamine plus 50 mg of zolazepam) reconstituted with 2.5 ml ketamine (100 g/L) and 2.5 ml xylazine (100 g/L) (Fort Dodge Animal Health, Fort Dodge, IA) and a dose of 0.03 ml/kg body weight following proven protocols (Keever et al., 2020). Following anesthetization, pigs were euthanized using an intracardial injection of sodium pentobarbital (86 mg/kg body weight, Fata Plus, Vortech Pharmaceuticals, Dearborn, MI), livers were dissected out, and samples were flash frozen in dry ice and stored at −80°C.

### Metabolomic Sample Preparation and Analysis

Individual samples were prepared following the protocol of Wu et al. (2008). Briefly, 50–150 mg of frozen tissue from individual pigs was placed in 8 ml/g cold methanol and 2.5 ml/g cold water and homogenized twice for 30 s. Additional 8 ml/g chloroform and 4 ml/g water were added before being vortexed for 60 s. The sample was left on ice for 10 min to allow the sample to partition into polar and non-polar layers. The sample was centrifuged for 10 min at 4C at 12,000 × g, and the “top layer” was removed for metabolite analysis.

Metabolite identifications and profiles were obtained at Metabolomics Center, Roy J. Carver Biotechnology Center, University of Illinois at Urbana-Champaign. A spectrum for each sample was acquired using a gas chromatography (GC)–mass spectrometry (MS) system (Agilent, Inc., CA, United States) consisting of an Agilent 7,890 gas chromatograph, an Agilent 5975 MSD, and an HP 7683B autosampler. Gas chromatography was performed on a ZB-5MS (60 m × 0.32 mm I.D. and 0.25 μm film thickness) capillary column (Phenomenex, CA, United States). The inlet and MS interface temperature was 250°C, and the ion source temperature was adjusted to 230°C. An aliquot of 1 μL was injected with the split ratio of 7:1. The helium carrier gas was kept at a constant flow rate of 2 ml/min. The temperature program was isothermal heating at 70°C for 5 min, followed by an oven temperature increase of 5°C/min to 310°C and a final 10 min at 310°C. The mass spectrometer was operated in a positive electron impact (EI) mode at 69.9 eV ionization energy in the m/z 30–800 scan range. Chromatogram peaks were identified using the Automatic Mass Spectral Deconvolution and Identification System (AMDIS) v2.71 (National Institute of Standards and Technology, MD) software and a custom-built MS database encompassing the National Institute of Standards and Technology (NIST) database entries. The MS search database included 460 unique metabolites. Known artificial peaks were removed, and all data were normalized to the internal standard (hentriacontanoic acid at 10 mg/ml).

### Statistical Analysis

Normality assumptions were evaluated, and the log-transformed (base 2) relative abundance of the detected metabolite was described using a mixed effect model that included the main effects of weaning, MIA, sex, and interactions and the random effects of gilt and pen (SAS Institute, Cary, NC, United States). The *p*-values from the tests were adjusted for multiple testing using the false discovery rate (FDR) criterion (Benjamini and Hochberg, 1995). Enrichment analysis used the MetaboAnalyst v4 software with default specifications (Chong et al., 2018; Chong et al., 2019; Chong and Xia, 2020). Findings are reported employing annotations from the Kyoto Encyclopedia of Genes and Genomes database (KEGG) human metabolic pathway (downloaded in October 2019) (Kanehisa and Goto, 2000; Kanehisa et al., 2019) and the Human Metabolome Database (Wishart et al., 2007; Wishart et al., 2009; Wishart et al., 2013; Wishart et al., 2018) and using identifiers from ChEBI (Hastings et al., 2016) and PubChem (Kim et al., 2019).

## Results

Across all pigs, there were 170 unique metabolites that were identified in at least one sample. The metabolite N-acetylglycine was detected in 20 out of the 24 weaned pigs yet was not detected in any of the 24 nursed pigs. Among the experimental factors tested, weaning stress presented significant effects in the highest number of metabolites.

### Effects of Weaning Stress on Metabolism Profiles

**FIGURE 1:**
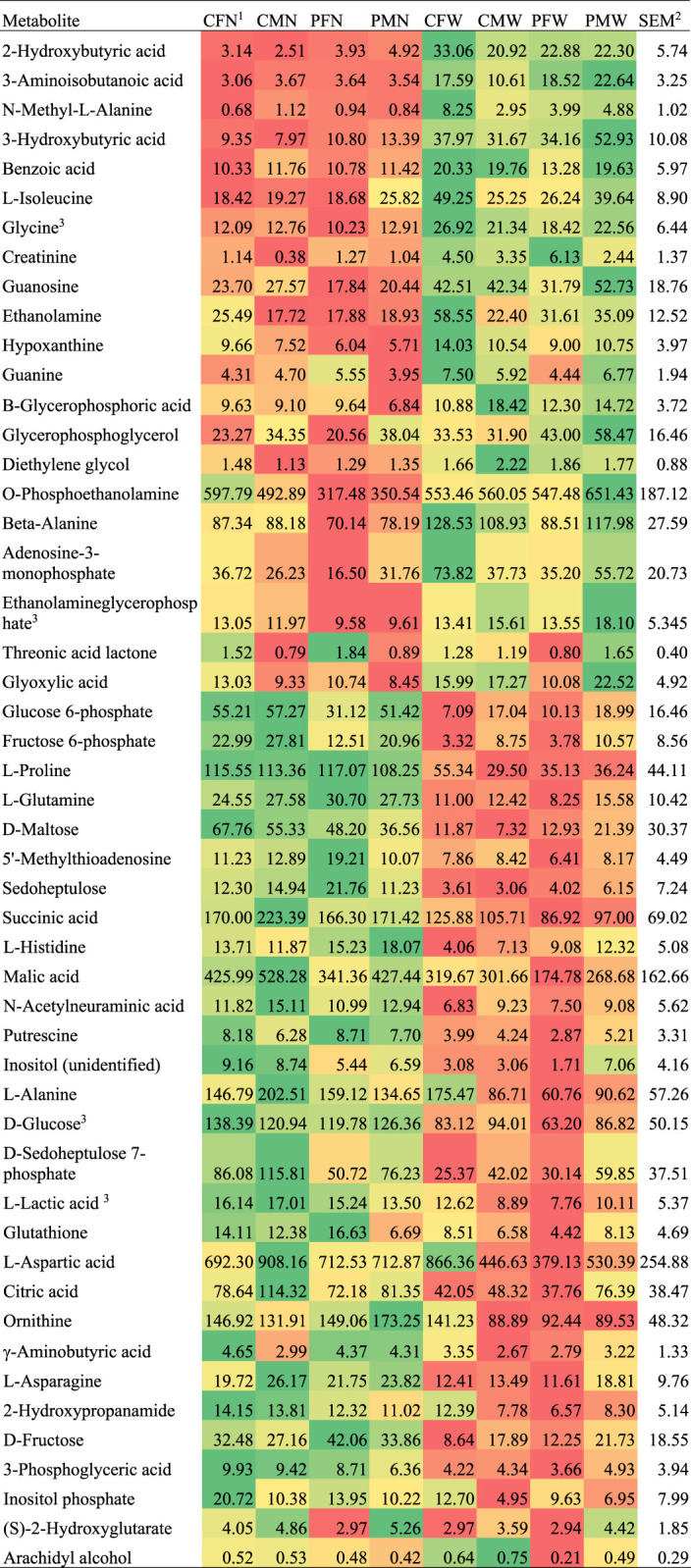
Abundance profile across weaning, maternal immune activation, and sex groups for metabolites that presented weaning effect alone or interacting with other factors.

A significant weaning effect at FDR-adjusted *p*-value < 0.05, corresponding to an unadjusted *p*-value < 0.01, was observed for 28 metabolites. An additional 10 metabolites had a significant effect of weaning alone or interacting with other experimental factors at FDR-adjusted *p*-value < 0.15, corresponding to an unadjusted *p*-value < 0.05. Table 1 lists the metabolites that had weaning effects, alone or interacting with MIA and sex, at *p*-value < 0.05. Figure 1 depicts the abundance profiles corresponding to the metabolites in Table 1 where red depicts low abundance and green depicts high abundance. Table 2 lists the KEGG pathways enriched at *p*-value < 0.05 (FDR-adjusted *p*-value < 0.2) and including at least three metabolites displaying weaning effects.

**TABLE 1:** Metabolites presenting at least one effect, weaning alone or interacting with maternal immune activation or sex, at *p*-value < 0.05.

		*p*-value of effect^1^						
Metabolite	Path^2^	W	M	S	MxS	MxW	WxS	WxMxS
(S)-2-Hydroxyglutarate	F	0.252	0.704	0.044	0.536	0.374	0.968	0.670
2-Hydroxybutyric acid	F	5.67E-12	0.097	0.378	0.264	0.192	0.693	0.484
2-Hydroxypropanamide	U	0.031	0.188	0.992	0.362	0.943	0.734	0.810
3-Aminoisobutanoic acid	A	8.81E-11	0.489	0.967	0.259	0.354	0.604	0.368
3-Hydroxybutyric acid	F	1.67E-06	0.320	0.556	0.153	0.555	0.917	0.650
3-Phosphoglyceric acid	C	0.042	0.865	0.997	0.502	0.362	0.622	0.982
5′-Methylthioadenosine	A	3.24E-04	0.910	0.548	0.165	0.327	0.139	0.170
Adenosine-3-monophosphate	P	0.028	0.423	0.526	0.035	0.417	0.853	0.496
Arachidyl alcohol	F	0.889	0.089	0.255	0.935	0.368	0.041	0.287
Benzoic acid	A	0.003	0.865	0.189	0.595	0.783	0.498	0.934
beta-Alanine	A	0.028	0.266	0.233	0.321	0.441	0.897	0.950
beta-Glycerophosphoric acid	F	0.014	0.865	0.595	0.430	0.783	0.055	0.592
Citric acid	C	0.018	0.744	0.095	0.582	0.160	0.682	0.530
Creatinine	A	2.57E-04	0.853	0.225	0.862	0.676	0.350	0.335
d-Fructose	C	0.034	0.303	0.843	0.821	0.561	0.171	1.000
d-Glucose	C	0.008	0.384	0.940	0.628	0.633	0.598	0.733
d-Maltose	C	1.41E-04	0.665	0.609	0.607	0.257	0.459	0.747
d-Sedoheptulose 7-phosphate	C	0.009	0.754	0.014	0.570	0.370	0.680	0.907
Diethylene glycol	F	0.022	0.932	0.995	0.908	0.723	0.441	0.420
Ethanolamine	F	0.008	0.660	0.353	0.159	0.442	0.846	0.921
Ethanolamine glycerophosphate	C	0.041	0.391	0.309	0.480	0.139	0.454	0.652
Fructose 6-phosphate	C	1.09E-06	0.583	0.004	0.501	0.352	0.132	0.881
gamma-Aminobutyric acid	A	0.026	0.382	0.278	0.153	0.649	0.347	0.691
Glucose 6-phosphate	C	1.75E-07	0.394	0.290	0.802	0.829	0.754	0.253
Glutathione	A	0.014	0.378	0.266	0.865	0.668	0.219	0.075
Glycerophosphoglycerol	F	0.018	0.273	0.032	0.244	0.166	0.195	0.666
Glycine	A	1.83E-04	0.420	0.494	0.367	0.707	0.811	0.865
Glyoxylic acid	A	0.001	0.288	0.458	0.053	0.508	0.031	0.405
Guanine	P	0.012	0.441	0.846	0.524	0.653	0.369	0.137
Guanosine	P	0.006	0.306	0.268	0.395	0.430	0.580	0.777
Hypoxanthine	P	0.009	0.332	0.942	0.563	0.531	0.892	0.883
Inositol (unidentified)	C	0.005	0.377	0.732	0.402	0.223	0.681	0.451
Inositol phosphate	U	0.048	0.483	0.233	0.192	0.261	0.763	0.721
l-Alanine	A	0.007	0.325	0.801	0.573	0.647	0.773	0.247
l-Asparagine	A	0.028	0.967	0.065	0.545	0.462	0.903	0.840
l-Aspartic acid	A	0.016	0.435	0.551	0.323	0.793	0.724	0.243
l-Glutamine	A	8.41E-05	0.892	0.292	0.961	0.792	0.177	0.522
l-Histidine	A	0.002	0.034	0.110	0.568	0.833	0.325	0.843
l-Isoleucine	A	0.004	0.503	0.449	0.086	0.935	0.692	0.615
l-Lactic acid	C	0.013	0.545	0.566	0.656	0.626	0.826	0.868
l-Proline	A	2.70E-06	0.772	0.875	0.796	0.583	0.998	0.556
Malic acid	C	0.003	0.218	0.140	0.627	0.761	0.920	0.859
N-Acetylneuraminic acid	C	0.003	0.983	0.242	0.271	0.683	0.876	0.622
N-Methyl-l-alanine	A	4.19E-09	0.508	0.754	0.662	0.967	0.699	0.256
O-Phosphoethanolamine	F	0.022	0.258	0.390	0.494	0.065	0.738	0.599
Ornithine	A	0.024	0.999	0.913	0.222	0.915	0.487	0.647
Putrescine	A	0.005	0.984	0.277	0.315	0.879	0.332	0.962
Sedoheptulose	C	0.002	0.703	0.508	0.829	0.682	0.197	0.138
Succinic acid	C	0.002	0.465	0.197	0.890	0.519	0.812	0.938
Threonic acid lactone	C	0.865	0.911	0.241	0.136	0.510	0.006	0.232

1 *p*-value for weaning (W), MIA (M), sex (S), interaction between MIA and sex (MxS), interaction between MIA and weaning (MxW), interaction between weaning and sex (WxS), and interaction between weaning, MIA, and sex (WxMxS). *p*-value < 0.01 = FDR-adjusted *p*-value < 0.05; *p*-value < 0.05 = FDR-adjusted *p*-value < 0.15.

^2^Metabolic pathway or classification: A = amino acid metabolism; C = carbohydrate metabolism; F = fatty acid metabolism; G = glycoprotein; P = purine and pyrimidine; U = unknown.

**TABLE 2:** Pathways enriched at *p*-value < 0.05 and including at least three metabolites among the molecules associated with weaning effects.

Database^1^	Category	Total^2^	Hits^3^	*p*-value	FDR^4^
MBDDB	Diabetes mellitus (MODY), non-insulin-dependent	19	7	3.4E-4	0.050
SMPDB	Warburg effect	58	8	0.001	0.099
SMPDB	Ammonia recycling	32	5	0.006	0.217
KEGG	Aminoacyl-tRNA biosynthesis	48	8	1.6E-5	0.001
KEGG	Alanine, aspartate, and glutamate metabolism	28	6	4.8E-5	0.002
KEGG	Starch and sucrose metabolism	18	5	5.7E-5	0.002
KEGG	Arginine biosynthesis	14	3	0.005	0.078
KEGG	Glyoxylate and dicarboxylate metabolism	32	4	0.008	0.108
KEGG	Amino sugar and nucleotide sugar metabolism	37	4	0.013	0.126
KEGG	Arginine and proline metabolism	38	4	0.015	0.126
KEGG	beta-Alanine metabolism	21	3	0.015	0.126
KEGG	Pentose phosphate pathway	22	3	0.017	0.131
KEGG	Propanoate metabolism	23	3	0.019	0.135
KEGG	Galactose metabolism	27	3	0.029	0.192
KEGG	Glutathione metabolism	28	3	0.033	0.196

1 MBDDB = MetaboAnalyst Blood Disease Database; SMPDB = Small Molecule Pathway Database; KEGG = Kyoto Encyclopedia of Genes and Genomes database.

2 Total number of metabolites in the category.

3 Hits = number of differentially abundant metabolites in the category.

4 FDR = false discovery rate–adjusted *p*-value.

The majority of the metabolites presented weaning effect acting independently from the other experimental factors. Among the metabolites over-abundant in weaned relative to nursed pigs in Table 1 and Figure 1 were beta-Alanine, adenosine-3-monophosphate, and ethanolamine glycerophosphate. Among the metabolites over-abundant in nursed relative to weaned pigs in Table 1 and Figure 1 were beta-Alanine, adenosine-3-monophosphate, ethanolamine glycerophosphate, gamma-aminobutyric acid, asparagine, 2-hydroxypropanamide (lactamide), fructose, 3-phosphoglyceric acid (3-phosphoglycerate), and inositol-p (inositol phosphate). Metabolites that presented a weaning-by-sex interaction effect or simultaneous sex and weaning effects included threonic acid, glyoxylic acid, arachidyl alcohol, glycerophosphoglycerol, d-sedoheptulose 7-phosphate, and (S)-2-hydroxyglutarate (Table 1).

Multiple metabolites exhibiting significant (FDR-adjusted *p*-value < 0.05) differential abundance between nursed and weaned pigs are annotated to the metabolism of carbohydrates, amino acids, purines, pyrimidines, and fatty acids (Table 1). Metabolites associated with energy metabolism such as alpha-d-glucose-6-phosphate and d-fructose-6-phosphate were significantly higher in nursed relative to weaned pigs. Significant lower levels of creatinine were observed in nursed relative to weaned pigs. Multiple glucogenic amino acids including alanine, aspartic acid, glutamine, histidine, and proline as well as intermediate amino acid metabolites were significantly higher in nursed relative to weaned pigs. However, glycine and isoleucine amino acids were significantly lower in nursed relative to weaned pigs. All compounds associated with fatty acid metabolism that had a significant (FDR-adjusted *p*-value < 0.05) association with weaning stress were detected at lower levels in nursed relative to weaned pigs.

The KEGG pathways enriched among the metabolites affected by weaning include aminoacyl-tRNA biosynthesis (Table 2). This pathway encompasses the processes of ligation between amino acids and tRNAs, and the metabolites l-glutamine and glycine. Also enriched were the pathways of alanine, aspartate and glutamate metabolism, arginine biosynthesis, glutathione metabolism, and glyoxylate and dicarboxylate metabolism (Table 1). The alanine, aspartate, and glutamate metabolism pathways encompass the differentially abundant metabolites l-glutamine, succinic acid, and l-aspartic acid.

### Effects of Maternal Immune Activation and Sex on Metabolic Profiles

**FIGURE 2:**
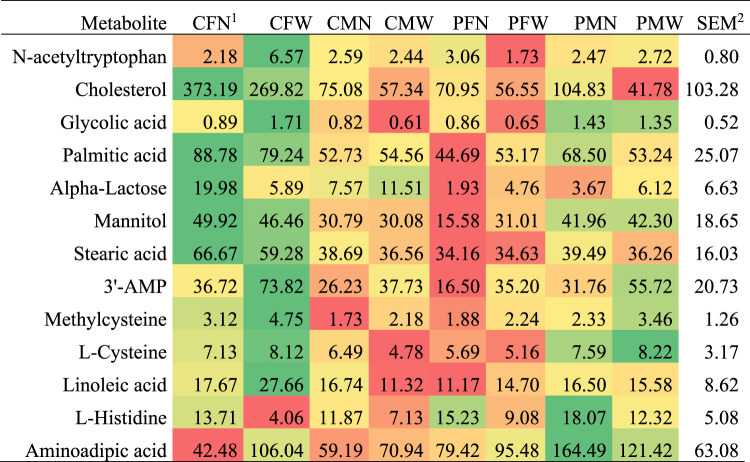
Abundance profile across weaning, maternal immune activation, and sex groups for metabolites that presented maternal immune activation effect alone or interacting with other factors.

Three metabolites presented MIA effects alone or interacting with weaning and sex at *p*-value < 0.01, and an additional 11 metabolites presented MIA effects at *p*-value < 0.05. Table 3 lists the metabolites that presented MIA effects, alone or interacting with sex, at *p*-value < 0.05, and Figure 2 depicts the corresponding abundance profiles. Table 4 lists the KEGG pathways enriched at *p*-value < 0.05 (FDR-adjusted *p*-value < 0.2) and including at least three metabolites among the molecules associated with MIA effects.

**TABLE 3:** Metabolites presenting at least one effect, maternal immune activation alone or interacting with weaning or sex, at *p*-value < 0.05.

		*p*-value^1^						
Metabolite	Path^2^	W	M	S	MxS	MxW	WxS	WxMxS
N-Acetyltryptophan	A	0.300	0.052	0.310	0.088	0.013	0.450	0.002
Cholesterol	F	0.300	0.003	0.076	0.069	0.790	0.470	0.230
Glycolic acid	F	0.390	0.880	0.520	0.006	0.930	0.430	0.380
Palmitic acid	F	0.720	0.150	0.590	0.019	0.940	0.900	0.140
alpha-Lactose	C	0.280	0.015	0.420	0.620	0.310	0.970	0.270
Mannitol	C	0.250	0.300	0.570	0.015	0.270	0.560	0.280
Stearic acid	F	0.850	0.100	0.200	0.021	0.910	0.730	0.490
3′-AMP	P	0.028	0.420	0.530	0.035	0.420	0.850	0.500
Methylcysteine	A	0.053	0.670	0.340	0.034	0.970	0.700	0.560
l-Cysteine	A	0.900	0.930	0.270	0.047	0.790	0.490	0.410
Linoleic acid	F	0.520	0.390	0.650	0.041	0.890	0.370	0.870
l-Histidine	A	0.002	0.034	0.110	0.570	0.830	0.330	0.840
Aminoadipic acid	A	0.380	0.046	0.270	0.350	0.350	0.620	0.820

1 *p*-value for weaning (W), MIA (M), sex (S), interaction between MIA and sex (MxS), interaction between MIA and weaning (MxW), interaction between weaning and sex (WxS), and interaction between weaning, MIA, and sex (WxMxS). *p*-value < 0.01 = FDR-adjusted *p*-value < 0.05; *p*-value < 0.05 = FDR-adjusted *p*-value < 0.15.

2 Metabolic pathway or classification: A = amino acid metabolism; C = carbohydrate metabolism; F = fatty acid metabolism; G = glycoprotein; P = purine and pyrimidine; U = unknown.

**TABLE 4:** Pathways enriched at *p*-value < 0.05 and including at least three metabolites among the molecules associated with maternal immune activation effects.

Source^1^	Category	Total^2^	Hits^3^	*p*-value	FDR^4^
SMPDB	Bile acid biosynthesis	65	3	0.044	>0.25
KEGG	Biosynthesis of unsaturated fatty acids	36	3	0.003	0.248

1 SMPDB = Small Molecule Pathway Database; KEGG = Kyoto Encyclopedia of Genes and Genomes database.

2 Total number of metabolites in the category.

3 Hits = number of differentially abundant metabolites in the category.

4 FDR = false discovery rate–adjusted *p*-value.

The outermost effects of MIA were observed for N-acetyltryptophan, cholesterol, and glycolic acid. Other metabolites presenting an MIA effect (*p*-value < 0.05) included 2-aminoadipic acid, stearic acid, palmitic acid, and methylcysteine. Most metabolites presented an MIA effect interacting with sex (e.g., glycolic acid) and fewer interacting with weaning (e.g., N-acetyltryptophan) or independent of other factors (e.g., adenosine-3-monophosphate, l-histidine). Both cholesterol and alpha-lactose had lower abundance in PRRSV-challenged pigs relative to control gilts (Table 3). For most metabolites, females presented the outermost differences between MIA groups. An even number of metabolites presented highest differences between MIA groups in nursed females (e.g., palmitic acid, stearic acid, mannitol) or in weaned females (e.g., l-cysteine, glycolic acid).

## Discussion

Pre- and postnatal stressors including weaning and maternal infection during gestation disrupt molecular signaling that can directly or indirectly affect hepatic function. Established effects of weaning on the HPA axis and of MIA on the developing offspring hippocampus and amygdala including inflammatory responses can disrupt metabolic processes in the liver. Likewise, the liver has a nervous system that senses lipids, glucose, and metabolite levels (after eating and drinking) and triggers the nervous system to make appropriate physiological changes. The liver performs sensor and effector roles, influencing and being influenced by neurological signals and ablation (Jensen et al., 2013). The immunocompetence of the hepatic cells develops during the prenatal and early postnatal periods and remains responsive during the life of the individual (Le Rouzic et al., 2011).

The present study employed GC–MS metabolomic analysis to uncover changes in liver molecules associated with both prenatal inflammatory stress and postnatal weaning stress. The metabolic signals from the more recent weaning stress were stronger than those from the farther-back inflammatory stress experienced during gestation. This study uncovered prolonged effects of inflammatory MIA on hepatic metabolites in addition to weaning effects and evidence of sex-dependency of these effects on some metabolic products.

### Effects of Weaning Stress on Metabolism Profiles

Weaning stress impacted the abundance metabolites across multiple metabolic pathways. Intermediate and end products of energy-related pathways, including carbohydrate and fatty acid metabolism, were prevalently disrupted (Moat et al., 2003b; Williams, 2008; Dashty, 2013; Harris and Harper, 2015; Adeva-Andany et al., 2016; Han et al., 2016). The metabolic changes associated with weaning reflect the rapid adaption of the liver to provide energy from different sources (Han et al., 2016). Similar changes in many energy-related metabolites and pathways were also observed in pigs 7 days after weaning (Xiao et al., 2012). These changes can be attributed to low feed intake immediately after weaning, and these effects can persist until approximately two weeks after weaning (Le Dividich and Seve, 2000).

The nutritional stress elicited by weaning is apparent in the presence of N-acetylglycine, an N-acetylated amino acid, in most of the weaned pigs, yet this metabolite was not detected in any of the 24 nursed pigs. Consistent with our findings, significantly higher levels of N-acetylglycine were associated with increased average dietary fiber intake (Lustgarten et al., 2014). Also, high levels of N-acetylated amino acids were linked to the aminoacylase 1 (ACY1) gene (Perrier et al., 2005; Van Coster et al., 2005), and feeding disruptions have been associated with ACY1 deficiency (Van Coster et al., 2005). The abundance of N-acetylglycine among weaned pigs detected in the present study may also reflect changes in diet because increased serum concentration of degraded amino acids has been reported with high protein intake (Burke et al., 2012), and N-acetylglycine is negatively associated with human body mass index (Moore et al., 2014).

One critical function of the liver is to transform energy sources into glycogen (Han et al., 2016). The 29 metabolites that were over-abundant in nursed relative to weaned pigs offered insights into the hepatic pathways that use milk-associated energy sources yet are not hindered by weaning stress. In response to increases in glucagon levels triggered by conditions such as fasting and hypoglycemia, hepatic glucose production is promptly stimulated via the gluconeogenesis process. In nursed pigs, the main energy source is glucose obtained by the degradation of maternal milk lactose by the intestines and potentially supplemented by creep feed sources available. The pathways enriched among the metabolites differentially abundant between weaned and nursed pigs in the present study confirm the transition of hepatic process in response to weaning (Table 1). Weaned pigs lack the lactose source and must transition to process solid feed as the energy source. Multiple energy pathways in the liver convert lactose and glucose sources into glycogen including the glycolysis/gluconeogenesis pathway, pentose phosphate pathway, and metabolism of amino acids, purines, pyrimidines, creatine, and fatty acids (Moat et al., 2003b; Moat et al., 2003a; Moat et al., 2003c; Williams, 2008; Dashty, 2013; Harris and Harper, 2015; Adeva-Andany et al., 2016; Han et al., 2016). Metabolites from the same pathways were detected in lower levels in the muscle of slow relative to fast growing pigs at 21 days of age (Ramsay et al., 2018). The shifts in hepatic metabolic pathways associated with weaning effects (Table 1, Figure 1) are consistent with metabolite changes detected in other tissues and organs in response to comparable conditions.

Multiple intermediate and final products of the enriched glycolysis and gluconeogenesis pathway (Table 1) that processes glucose into pyruvate were differentially abundant between weaned and nursed pigs. The levels of glucose 6-phosphate and fructose 6-phosphate were significantly higher in nursed relative to weaned pigs (Table 1, Figure 1), and this pattern is consistent with reports of weaning effects in pigs (Le Dividich and Seve, 2000). Another metabolite of the glycolysis pathway, 3-phosphoglyceric acid, exhibited a consistent yet lesser extreme profile than glucose and fructose. Our results indicate that the highest impact of weaning on hepatic gluconeogenesis metabolism occurs in the priming stage that culminates with fructose 1,6-bisphosphate (Harris and Harper, 2015).

In addition to glucose 6-phosphate and fructose 6-phosphate, other metabolites pertaining to the enriched pentose phosphate pathway (Table 1) were differentially abundant between weaned and nursed pigs. An example is sedoheptulose, a metabolite that can be processed into pentose, and following the glucose and fructose patterns, the levels of sedoheptulose and sedoheptulose 7-phosphate were significantly higher in nursed relative to weaned pigs (Table 1, Figure 1). The level of sedoheptulose 7-phosphate was also higher in males than in females. The established effect of 7-0-galloyl-d-sedoheptulose protecting hepatic tissue from oxidative stress, inflammation, and apoptosis suggests that weaned pigs are likely to be more susceptible to these processes (Noh et al., 2012).

Metabolites that can be processed into glycogen were detected at significantly higher levels in nursed relative to weaned pigs. These metabolites included d-maltose (processed into 2-d-glucose), d-glucose (processed into alpha-d-glucose-6-phosphate), d-fructose (converted into d-fructose-6-phosphate), lactic acid (converted to pyruvate), citric acid, succinic acid, and malic acid. The levels of two inositol metabolites, inositol-p and an unidentified form of inositol, were also significantly higher in nursed relative to weaned pigs. Inositol processes and metabolic products play critical roles in cardio-metabolic diseases and modulate diet-induced obesity, diabetes, fatty liver, and bacterial infection (Chakraborty, 2018).

Glucogenic amino acids can be processed into glucose in the liver, and the levels of multiple molecules in this category were significantly higher in nursed relative to weaned pigs including proline, glutamine, alanine, asparagine, aspartic acid, and histidine (Table 1, Figure 1). The lower levels of histidine in weaned pigs may indicate an impaired immune response because histidine is a precursor for histamine (Moya-Garcia et al., 2005; Schneider et al., 2010), although histidine also presented MIA effects. Likewise, within the enriched pathway of arginine and proline metabolism, responsible for the biosynthesis of these amino acids from glutamate, the levels of the metabolites putrescine and ornithine were higher in nursed relative to weaned pigs (Table 1).

Several intermediate products of the amino acid metabolic pathways were affected by weaning stress. gamma-Aminobutyric acid participates in the metabolic pathways of many amino acids, and consistent with the amino acid levels previously reviewed, the level of gamma-aminobutyric acid was higher in nursed relative to weaned pigs. Within the amino acid metabolic pathways of cysteine and methionine, the levels of both 5′-methylthioadenosine and glutathione were higher in nursed relative to weaned pigs. In addition to the role in the amino acid pathway, glutathione plays a role in antioxidant immune defense, and detected profiles indicate that weaned pigs may have compromised immune defense (Lu, 2009).

N-Acetylneuraminic acid was over-abundant in nursed relative to weaned pigs. This metabolite belongs to the group of cell surface glycans involved in molecular interactions (Schauer, 2009), and glycoproteins participate in immune response (Schauer, 2009; Moons et al., 2019; Xiong et al., 2019), especially in the mucus protective barrier (Corfield, 2018). Impaired intestinal barrier functions against noxious antigens and pathogens are associated with low or no feed intake that occurs immediately after weaning (Wijtten et al., 2011). The difference in N-acetylneuraminic acid between nursed and weaned pigs can be associated with changes in gut health after weaning, in response to changes in intake and stress conditions that impact the immune system (Moeser et al., 2017; Pluske et al., 2018).

The 21 metabolites that were over-abundant in weaned relative to nursed pigs offered insights into the hepatic pathway shift triggered by milk no longer being the sole source of energy and weaning stress impacting the immune and endocrine systems. The levels of the amino acids glycine and isoleucine were lower in nursed relative to weaned pigs (Table 1, Figure 1), and this profile is consistent with the deficiency of N-acetylglycine in nursed pigs. The difference between weaned and nursed pigs is not likely due to changes in diet because while porcine milk contains endogenous amino acids, dietary amino acids are initially catabolized in the small intestine by mammalian enterocytes or bacteria (Hou et al., 2016). The amino acid patterns observed in the present study are in agreement with reports of low postweaning protein accretion (Le Dividich and Sève, 2001).

Among the differentially abundant molecules in the enriched alanine pathway (Table 1), the levels of N-methyl-l-alanine (a metabolite that can be processed into pyruvate) and benzoic acid were lower in nursed relative to weaned pigs. This pattern may be associated with the frequently observed increase in amino acid metabolism in the presence of excess amino acids (Barber et al., 1990; Gall et al., 2010).

The levels of creatine, a member of the arginine and proline metabolic pathway, were lower in nursed relative to weaned pigs. The higher level of creatinine in 22-day-old weaned relative to nursed pigs is consistent with the difference in creatinine serum levels between pre- and postweaning seven-day-old pigs (Xiao et al., 2012). As an energy source, creatine is mainly produced by the liver from arginine, glycine, and methionine, and creatinine is a byproduct of the corresponding metabolic pathways (Wyss and Kaddurah-Daouk, 2000). A small proportion of creatinine in suckled pigs can be attributed to approximately one-fourth of the creatine provided by milk (Brosnan et al., 2009). However, the lower levels of creatinine in nursed relative to weaned pigs may reflect the higher use of creatine in the weaned pig as an additional energy source.

The amino acid glutathione anabolic pathway encompasses 2-hydroxybutyric acid (butanoic acid, 2-hydroxy), and this molecule was lower in nursed relative to weaned pigs. Similarly, beta-aminoisobutyric acid is a non-protein amino acid that originates from the catabolism of thymine and valine, and the pattern of this amino acid was consistent with that of 2-hydroxybutyric acid. This profile and the known role of butanoic acid further confirm that weaned pigs experience oxidative stress in the liver (Gall et al., 2010).

Nucleotide metabolism is an important energy source in animals (Moffatt and Ashihara, 2002; Moat et al., 2003c). Most of the purine (i.e., guanosine, guanine, hypoxanthine) and pyrimidine (beta-alanine) metabolites were detected at significantly lower levels in nursed relative to weaned pigs. On the contrary, l-glutamine, an intermediate product of purine and pyrimidine metabolism, was more abundant in weaned than in nursed pigs. The previous patterns suggest that weaned pigs are using purine and pyrimidine nucleotides as an additional energy source, while nursed pigs use other more readily available and efficient energy sources provided by the maternal milk.

The levels of multiple metabolites associated with fatty acid metabolism were significantly lower in nursed relative to weaned pigs, such as O-phosphoethanolamine, ethanolamine (participating in glycerophospholipid metabolism), 3-hydroxybutyric acid (participating in ketone metabolism), glycerophosphoglycerol (the glycerolipid glycerol ester of glycerophosphoric acid), and beta-glycerophosphoric acid (participating in glycerolipid metabolism). Also, the level of glycerophosphoglycerol was also higher in males than in females (Table 1). The observed patterns of fatty acid oxidation products are in agreement with a higher level of 3-hydroxybutyric acid in fasting or malnourished patients (Kawaguchi et al., 2008).

### Effects of Maternal Immune Activation on Metabolic Profiles

The activation of the immune response of the mother in response to viral infection during gestation and subsequent impact in the offspring was associated with changes in metabolic products (Table 3, Figure 2). While weaning effect influenced the profile of a larger number of metabolites, with a stronger and independent effect on the molecules, the effects of MIA were on fewer metabolites, milder, and interacting with other experimental factors. Our finding of sex-dependent MIA effects on liver metabolites is consistent with a report that MIA elicited by gestational exposure to the toxic agent titanium dioxide was associated with newborn hepatic differential gene expression in females but not in male mice (Jackson et al., 2013).

Changes in the levels of intermediate and end products of energy-related pathways, including carbohydrate and fatty acid metabolism, were associated with MIA. Tryptophan is a glucogenic amino acid, and the processing of this molecule into glucose occurs in the liver. The level of N-acetyltryptophan was significantly lower in PRRSV-challenged weaned pigs relative to control gilts (Table 1, Figure 1). Presenting an opposite profile, the glucogenic amino acid histidine was more abundant in PRRSV-challenged pigs relative to control gilts. The apparent inconsistent profiles of the glucogenic amino acids histidine and acetyltryptophan in response to MIA may be due to the role of histidine in inflammation, and this role may supersede the role as an energy source. Histidine is a precursor for histamine, and the histidine profile observed in the present study suggests chronic hepatic inflammatory priming due to MIA (Moya-Garcia et al., 2005; Schneider et al., 2010). Our finding of the effect of MIA on hepatic immune response supports reports that maternal immune activation can affect various hepatic inflammatory mediators in the liver of the offspring (Jackson et al., 2013).

Cholesterol is an important component of membrane bilayers, and abnormal levels are detrimental to cell function (Cohen, 2008). The lower levels of cholesterol detected in PRRSV-challenged pigs relative to control gilts indicate that MIA may cause hypocholesterolemia. Serum hypocholesterolemia has been associated with autism spectrum disorder, a neurodevelopmental and behavioral disorder linked to MIA (Benachenhou et al., 2019).

The level of 2-aminoadipic acid was higher in PRRSV-challenged pigs relative to control gilts. As a product of the lysine biosynthesis and degradation pathways, increased levels of 2-aminoadipic acid were detected in the liver of mouse experiencing gravitational stress relative to controls (Pecaut et al., 2017). The metabolite 2-aminoadipic acid contributes to the regulation of glucose homeostasis (Wang et al., 2013), and high levels are indicative of oxidative stress (Zeitoun-Ghandour et al., 2011; Pecaut et al., 2017; Ou et al., 2019). Also, 2-aminoadipic acid has been associated with immune and metabolic liver diseases (Cichoz-Lach and Michalak, 2014) including type 2 diabetes (Wang et al., 2013; Lees et al., 2017).

The level of lactose was lower in PRRSV-challenged pigs relative to control gilts. The lactose detected in the liver is likely an immediate product from the galactose pathway because lactose from the milk is converted into d-glucose and d-galactose by the small intestine. The pattern detected in the present study suggests that MIA may interfere with steps of the galactose pathway that process lactose into final products.

Metabolic changes due to MIA stress on hepatic metabolism support the development of treatments to ameliorate the effects of this stressor. For example, high-fat, low-carbohydrate ketogenic diets have been linked to low inflammatory profiles and are used to ameliorate comorbidities of MIA-associated autism spectrum disorder (Ruskin et al., 2017). Also, altering dietary protein and carbohydrate intake can induce the nervous system to rely on pathways using liver-generated ketone bodies (such as acetoacetate and β-hydroxybutyrate, acetone) for energy sources instead of using other metabolites such as histidine that are used in the immune response.

## Conclusion

Our work advances the understanding of the effects of weaning stress and MIA that can affect hepatic metabolism and influence health and growth. Weaning stress can elicit molecular signals in the central and peripheral nervous and gastrointestinal systems in humans, rodents, and pigs. The maternal response to infection during gestation can also elicit inflammatory responses known to affect neurodevelopmental processes. Conversely, products of the liver metabolism can influence pathways in the amygdala, the hippocampus, and other brain regions.

The enriched glycolysis and gluconeogenesis, pentose phosphate, and amino acid metabolic pathways included many metabolites that were over-abundant in nursed relative to weaned pigs. The enriched pathways confirm the impact of weaning on hepatic metabolic shift, oxidative stress, and inflammation. Creatine and several metabolites annotated to the alanine, arginine, and proline pathways were over-abundant in weaned relative to nursed pigs. The previous patterns reflect the higher use of amino acids in the weaned pig as an additional energy source.

Our findings can assist in the identification of management that minimizes hepatic dysfunction associated with weaning and gestational stressors. The level of the glucogenic metabolite N-acetyltryptophan was lower in PRRSV-challenged weaned pigs relative to control gilts, whereas the level of another glucogenic amino acid histidine presented the opposite profile. The histidine profile reflects the role of this metabolite in modulating inflammation. The profiles of ketone, carbohydrates, fatty acids, and other metabolic products characterized in this study can aid in further enhancements of treatments to minimize the effects of weaning and MIA.

## Data Availability

The dataset generated for this study can be found in the Illinois Data Bank https://doi.org/10.13012/B2IDB-9043394_V1.
